# The Energy Saving and Emission Reduction Effect of Carbon Trading Pilot Policy in China: Evidence from a Quasi-Natural Experiment

**DOI:** 10.3390/ijerph19159272

**Published:** 2022-07-28

**Authors:** Huan Zhang, Jingyu Wu

**Affiliations:** 1 School of Economics, Nanjing Audit University, Nanjing 211815, China; 270336@nau.edu.cn; 2 Reading Academy, Nanjing University of Information Science and Technology, Nanjing 210044, China

**Keywords:** emissions reduction, energy conservation, carbon emission trading, policy effect, carbon neutrality

## Abstract

Promoting the carbon emission trading system has been a crucial measure for China to fulfill its carbon neutrality commitment. Taking the carbon emission trading system implemented in China in 2013 as a quasi-natural experiment, based on the provincial panel data of China from 2005 to 2019, this paper adopts the difference-in-difference (DID) method and the synthetic control method (SCM) to evaluate the impact of the carbon emission trading system on energy conservation and emission reduction in pilot provinces and cities. The research findings reveal that, on the whole, the carbon emission trading system has significantly promoted the process of energy conservation and emission reduction in pilot provinces and cities. Other robustness tests, including the parallel trend test, PSM–DID stationarity test and placebo test have also been passed. Heterogeneity analysis shows that the most significant policy effects occur in Tianjin and Shanghai, followed by Hubei. The emission reduction effect of Guangdong displays a trend of first decreasing and then increasing. The test results demonstrate that the carbon emission trading system can strengthen the process of energy conservation and emission reduction by optimizing the industrial structure and energy structure. In conclusion, policy makers should coordinate the relationship between the government and the market and speed up the transformation of environmental policy from command control type to market incentive type. Meanwhile, improve the property right system and accelerate the promotion of carbon emission trading pilot policies in China according to local conditions. By encouraging technological innovation, a new market-oriented path of energy conservation and emission reduction guided by the enhancement of energy efficiency and the optimization of energy and industrial structures ought to be formed.

## 1. Introduction

The problem of global warming is becoming increasingly serious, mainly due to the rise in temperature caused by the surge in carbon emissions. While China’s economy has developed rapidly for decades, it has also produced a series of social issues. Among them, environmental problems are major concerns that need to be solved and related to people’s livelihoods [1]. The escalating global carbon emissions have led to a progressively stronger greenhouse effect, which arouses substantial concern in the international community. China’s carbon emissions account for about 30% of the world total, far exceeding that of European and American countries, so it needs to bear greater responsibility for emission reduction. Therefore, the government has formulated a series of emission reduction targets, emphasizing the adoption of more powerful policies and countermeasures to strive to peak carbon dioxide emissions by 2030 and achieve carbon neutrality by 2060. Controlling energy consumption and reducing carbon emissions are the key to the low-carbon transformation of economy and society. In order to achieve these carbon emission reduction targets, effectively respond to global climate change, promote green and low-carbon development, and accelerate the transformation of economic development modes and the upgrading of industrial structure, China has to explore the carbon trading market mechanism to deal with the greenhouse effect and environmental problems [2]. To cope with climate change, the world’s carbon emission trading system was first established in the EU in 2005. As a large developing country, China attaches great importance to atmospheric pollution and actively investigates the utilization of market means to curb greenhouse gas emissions.

Being a crucial institutional innovation to control carbon dioxide emissions aided by the market mechanism, China has regarded the construction and improvement of carbon emission trading market as an indispensable policy tool for reaching peak emissions in the government work report of the 14th Five-year Plan. China’s carbon emission trading policy began with the *Notice on Carrying out the Pilot Work of Carbon Emission Trading* issued by the National Development and Reform Commission in 2011, which specifies to launch the pilot work of carbon emission trading in seven provinces and cities: Beijing, Shanghai, Hubei, Chongqing, Guangdong, Tianjin and Shenzhen. This policy considers carbon emissions as a commodity, uses varied emission reduction costs among enterprises to form a price difference, and is intended to realize the effective allocation of emission reduction resources through the market mechanism to achieve the goal of low-cost emission reduction. The government advocates the pilot work of carbon emission trading and applies market means to encourage enterprises to engage in green technology innovation and future carbon emissions abatement, which is the target of the carbon trading pilot policy. In actual practice, due to the heterogeneity of institutional backgrounds and technological innovation levels, carbon emission trading will have significant differences in terms of the emission reduction effect and the “Porter effect” [3].

Currently, the national carbon trading market has been officially launched, which signifies that China’s national carbon trading has opened the prelude. Carbon emission trading has become a vital way for China to deal with the problem of energy conservation and emission reduction. As the world’s largest carbon emitter, facing the pressure of domestic climate governance and international responsibility for emission reduction, after integrating the early pilot experience, China officially began to establish a national carbon trading mechanism at the end of 2017. The pilot policy of carbon emission trading has achieved an obvious emission reduction effect in China [4]. However, the construction of China’s carbon trading mechanism is still in its infancy, and the impact of the mechanism on carbon emissions is lack of in-depth quantitative analysis. As a means of market incentive environmental regulation, whether the pilot policy can advance the development of a regional green economy through energy saving and emission controls is worthy of further discussion. In view of this, the paper systematically analyzes the energy saving and emission reduction effects of carbon trading pilot policies by comparing the carbon emissions in pilot areas and non-pilot areas. The main objectives of the research are reflected in three fields: (1) through the overall and long-term dynamic effect test, to observe the evolution process of the carbon trading pilot policy on energy conservation and emission reduction; (2) probe the impact mechanism from the perspective of energy utilization efficiency; (3) according to the impact characteristics of different pilot areas, to customize the policy guidance programs for green development in each region.

The importance of this study is that the findings will provide a reference for enhancing the green development of the regional economy, which is beneficial to the formulation of carbon trading policies and the realization of emission reduction targets. The remainder of this paper is arranged as follows. The second section is a literature review, consisting of literature classification and summarization in related fields. The third section introduces the research method, which expounds the energy conservation and emission reduction model and policy evaluation model, as well as explaining the sample data. The fourth section displays the empirical results and discussion, comprising the existence test of the effect, quantitative analysis, energy saving and emission reduction effect analysis of carbon trading policy. The last section exhibits the conclusions and policy recommendations.

## 2. Literature Review

### 2.1. Technological Innovation Effect of Environmental Policy

At present, there are three strands of viewpoints on the research into environmental policy and technological innovation. The first argument holds that the “Porter Hypothesis” indeed exists, which holds that environmental regulation can promote technological innovation. For now, there are numerous scholars supporting the “Porter Hypothesis”. Qi et al. [5] employed the panel data of listed companies to build a DDD (Difference-in-Difference-in-Difference) model to study whether the environmental equity trading market can bring about green innovation. The results suggest that the policy can induce green technology innovation activities in polluting enterprises in the pilot area. Cao et al. [6] took environmental regulation as an exogenous variable and endogenous variable in separate analyses; it is found that the findings are consistent. Environmental regulation has a significant role in catalyzing green technology innovation, and there are obvious policy and regional differences. Bernard et al. [7] used the two-stage analysis method to study the relationship between environmental regulation and R&D (research and development) investment, and discovered that there was a positive correlation between them. Environmental regulation can encourage enterprises to increase R&D investment and induce technological innovation. The second argument opposes the existence of the “Porter Hypothesis”. Neoclassical economists believe that excessively strict environmental regulation will increase the cost and reduce the profit of enterprise, weaken investment in technological innovation, and inhibit technological innovation [8]. The study of Orugba et al. [9] concluded that whether environmental regulation is strict or not, local regulations have no impact on green technology innovation. Collecting the micro companies as samples, Dutta [10] and Anke et al. [11] proved that EU (European Union) carbon trading plays a very trivial role in low-carbon technology innovation. Carbon trading in the EU may lead to high emission technology innovation [12]. The third argument holds that there is a nonlinear relationship between environmental regulation and innovation. Based on the study of Hossain and Farooque [13] and Zhao et al. [14], the impact of environmental regulation on technological innovation is conditional. Through empirical analysis, Chen et al. [15] asserted that there is a U-shaped relationship between environmental regulation and green technology progress. It was revealed that there is a U-shaped relationship between the intensity of environmental regulation and technological innovation. The research on the central and western regions does not support the “Porter Hypothesis”, but the “Porter Hypothesis” has been verified in the eastern region [3].

### 2.2. Carbon Emission Reduction Effect of Carbon Trading Policy

In theory, a carbon trading system can clarify the property rights of carbon emission rights and achieve the purpose of total carbon emission control. However, due to the differences in green technology means, environmental system conditions and the construction degree of the carbon trading market, the evaluation results of the carbon emission reduction effect of the carbon trading policy are inconsistent [16,17].

On the one hand, studies by most scholars have concurred that the implementation of a carbon trading policy can play a positive role in carbon emission reduction. Based on the practical evidence of the construction of the German carbon trading market, it is reflected that the carbon trading policy plays a significant role in promoting social emission reduction [18,19]. The EU carbon trading mechanism confirmed that the carbon market strictly limits pollution, thus contributing to carbon emission reductions [20,21,22]. India’s carbon market research suggests that carbon trading significantly inhibits carbon emissions, especially the emission reduction effect on the power generation industry [23]. Similarly, the construction of an international carbon trading market can effectively curb global carbon emissions [24,25]. With regard to the research on China, the evaluation results of carbon emission reduction effects of a carbon trading pilot policy indicate that the carbon trading system can effectively promote emission reductions [26,27,28]. Lin and Jia [29] further pointed out that the emission reduction effect of carbon trading policies is gradually strengthened in the time dimension.

On the other hand, some scholars deem that the emission reduction effect of carbon trading policy is uncertain or insignificant. The effects of carbon trading policies show significant differences in the time dimension, in which it is difficult to effectively exert the inhibitory effect of carbon emission reduction in the short term, which aggravates the uncertainty of the effect of policy implementation [30]. Focusing on the seven carbon trading pilot provinces and cities established by China, it is concluded that the emission reduction effects of the pilot areas are widely different, and the pilot areas such as Tianjin and Chongqing have not achieved the expected effect of carbon emission reduction [31]. The above conclusion is supported by the research of Xuan et al. [32]. In addition, the evaluation results of the emission reduction effect of China’s emission trading system display that the emission trading system may not play a substantive role in social emission reduction at the present stage [33,34]. Therefore, this study proposes the first research hypothesis.

**Hypothesis** **1** **(H1).** *The carbon trading pilot policy can realize a low-carbon economy by reducing carbon emissions.*

### 2.3. Impact of Carbon Trading Policy on Energy Efficiency

As an approach to environmental regulation in the system reform, the relationship between carbon emission trading systems and energy efficiency has been widely debated. The research based on the “Porter Hypothesis” holds that environmental equity instruments can stimulate innovation, generate an innovation compensation effect and raise energy efficiency. Based on the panel data of 30 provinces in China, the empirical study found that environmental regulation and environmental innovation have a direct, positive effect on energy efficiency [35], which is consistent with the research results of Lin and Jia [36] through case analysis. Some other scholars believe that there is a nonlinear relationship between environmental regulation and energy efficiency, mainly due to the two natural attributes of “scarcity” and “externality” of energy, which makes the leading force affecting energy efficiency evolve from the “innovation compensation effect” to the “cost following effect” [37]. Moreover, from the perspective of energy, some scholars claim that there is an “energy rebound effect” [38]. That is, although technological progress can advance energy efficiency and realize the effect of energy conservation, it will also reduce the unit production price and cost of products, further increasing the actual demand for and consumption of products, which eventually bring more energy consumption, resulting in additional energy consumption offsetting the energy saved by energy efficiency improvement. Under the dual constraints of ecological environment and energy supply, enterprises will make full use of the carbon emission trading market mechanism to promote energy efficiency [39,40]. Enterprises with high energy efficiency will sell excessive quotas to obtain additional income, and thus further uplift energy efficiency based on low-carbon technological innovation. Enterprises with low energy efficiency will take the initiative to save energy, reduce emissions in the production process and elevate energy efficiency due to survival pressure and lack of low-carbon technology [41].

The carbon emission trading mechanism also has an important impact on energy consumption. The design of different carbon trading systems has an important influence on the emission reduction technology innovation process of the German power industry, which makes the fuel used for power generation convert from high carbon emission fuel to natural gas, and the energy consumption structure changes accordingly [42]. Through the establishment of CGE (Computable General Equilibrium) model to simulate and analyze the carbon trading policy, the inhibitory effect of carbon trading policy on energy demand is not strong, and the energy saving effect is not obvious in the short term [43,44]. Under the restrictions of carbon emission quotas, the emission reduction measures that enterprises may adopt consist of lessening output and strengthening technological innovation [45]. Technical progress may not only boost the energy efficiency of enterprises, but also uplift the utilization rate of low-carbon clean energy such as natural gas, hydropower and nuclear power, and reduce the utilization rate of high-carbon energy such as coal [46]. Enhancing energy efficiency is conducive to reducing total energy consumption. Raising the utilization rate of clean energy is advantageous to optimization of the energy consumption structure [47]. Therefore, this study proposes the second and third research hypotheses.

**Hypothesis** **2** **(H2).** *The carbon trading pilot policy facilitates energy intensive utilization.*

**Hypothesis** **3** **(H3).** *The carbon trading pilot policy helps achieve energy conservation and emission reduction by improving energy efficiency.*

### 2.4. Current Literature Gap and Contribution of This Study

Most of the existing literature focuses on carbon trading and emission reduction or carbon trading and energy efficiency, and few studies consolidate them into one analytical framework. The current literature only concentrates on the carbon emission reduction effect of carbon trading, and fails to effectively uncover the feasible path of carbon trading policy to attain energy conservation and emission reduction. Due to the short introduction time of China’s carbon trading pilot policy, there is still a relative lack of empirical research in this regard. However, there exists an obvious lag in the research compared to the policy planning of China’s full implementation of carbon emission trading market. Considering that the ultimate goal of the carbon trading policy is to realize energy-efficient utilization and the reduction of carbon dioxide emissions, it is necessary to accurately evaluate the energy saving and emission reduction effect of the carbon trading pilot policy, so as to provide empirical support for China to launch the national carbon emission trading market.

In view of this, based on the provincial panel data of China from 2005 to 2019, this paper uses the difference-in-difference (DID) method to empirically verify whether the pilot policy of carbon emission trading has achieved energy conservation and emission reduction. Compared with the extant literature on the energy saving and emission reduction effects of China’s carbon trading, the contributions of this paper mainly lie in the fields listed below. First, employing empirical data and the DID model, this paper empirically examines the environmental effects of the carbon emission trading pilot policy implemented in China, which provides direct empirical evidence for the implementation effect of the policy and enriches the research findings in this field. Second, this paper not only validates the impact of the carbon emission trading policy, but also further analyzes the efficiency mechanism of the carbon emission trading policy to attain energy conservation and emission reduction. That is to say, it investigates if the policy can achieve energy saving and emission control by adjusting the energy consumption structure and optimizing energy efficiency. Third, the research conclusions of this paper can provide empirical support and policy suggestions for China to establish and enhance the carbon emission trading system and implement the national unified carbon emission trading market.

## 3. Methodology and Data

### 3.1. Baseline Regression Model

This paper uses the difference-in-difference (DID) model to evaluate the emission reduction effect of the carbon emission trading policy. This model can control the ex ante differences of research objects and effectively separate the policy effect from other influencing factors [48]. Therefore, it is often used in the empirical research of policy evaluation. This paper selects 2013 as the impact point of the pilot policy of carbon trading market. Utilizing the nature of “quasi-natural experiment” of the new policy, FE–DID (fixed effect with difference-in-difference) can solve endogenous problems as much as possible, reduce the deviation of omitted variables and improve the accuracy of policy effect evaluation. In order to study the average policy effect of carbon trading on energy conservation and emission reduction, this paper constructs the following model.

(1) $$Y_{it}=\alpha_{0}+\alpha_{1}Pilot_{i}\times Time_{t}+\overset{6}{\underset{k=1}{\sum }}\beta_{k}Control_{k}+\gamma_{i}+\lambda_{t}+\varepsilon_{it}$$

where i and t represent province i and year t respectively; *Y_it_* represents the energy conservation and emission reduction of province i in year t. *Pilot_i_* is a policy dummy variable. If Province i is a pilot province of carbon trading, then *Pilot_i_* = 1; otherwise, it is 0. *Time_t_* is a dummy variable of the year, and 2013 is the year of policy impact, thus when t ≥ 2013, *Time_t_* = 1; otherwise, it is 0. *α*_1_ is used to estimate with DID, indicating the impact of carbon trading on provincial carbon emission reduction and energy saving. *Control* represents the control variables matrix, *ε* is a random disturbance term, *γ* and *λ* represent the province and time fixed effects respectively.

### 3.2. Dynamic Regression Model

In this paper, multiple interactive terms are added to extend the model of Formula (1) to further verify the dynamic policy effect of carbon trading on emission reduction year by year:

(2) $$Y_{it}=\alpha_{0}+\alpha_{t}\overset{2019}{\underset{t=2013}{\sum }}Pilot_{i}\times Time_{t}+\overset{6}{\underset{k=1}{\sum }}\beta_{k}Control_{k}+\gamma_{i}+\lambda_{t}+\varepsilon_{it}$$

where, *α*_1_ represents the net impact of carbon trading on energy conservation and emission reduction of provinces in year t, and the other variables are the same as Formula (1); *α*_0_, *β_k_*, *γ_i_*, *λ_t_* are parameters to be estimated.

### 3.3. Synthetic Control Method (SCM)

In this paper, the synthetic control method is adopted to estimate the policy effect of carbon trading pilot. According to the method of Abadie et al., (2010), it is assumed that there are *j* + 1 sample provinces, of which the first province is a pilot province of carbon trading, and the other J provinces are non-pilot provinces.

The sample has a total of *T* periods. *T*_0_ represents the period determined as the pilot province of carbon trading, in which 1 ≤ *T*_0_ ≤ *t*. $E_{it}^{I}$ represents the carbon emission and energy utilization of province *i* under the intervention of the pilot policy at time *t*, and $E_{it}^{N}$ represents the carbon emission and energy utilization of province *i* without the intervention of the pilot policy at time *t*. Therefore, the impact of carbon trading pilot policy on energy conservation and emission reduction can be expressed as $\alpha_{it}=E_{it}^{I}-E_{it}^{N}$. Let $E_{it}$ be the actually observed carbon emission and energy utilization of province *i* at time *t*, and $E_{it}=E_{it}^{I}$ under the influence of the pilot. If only the first province is assumed to be the pilot province of carbon trading, then after the pilot, i.e., *t* > *T_0_*, the policy effect is $\alpha_{1t}=E_{1t}^{I}-E_{1t}^{N}=E_{1t}-E_{1t}^{N}$. However, $E_{1t}^{N}$ is unobservable and needs to be estimated. The estimation model of $E_{it}^{N}$ can be expressed as:

(3) $$E_{it}^{N}=\delta_{t}+\theta_{t}Z_{i}+\lambda_{t}\mu_{i}+\varepsilon_{it}$$

where *Z_i_* is the observable predictive variable that is not affected by the policy, $\delta_{t}$ represents the time trend, $\theta_{t}$ represents the unknown parameter, $\lambda_{t}$ represents the unobservable common factor, $\mu_{i}$ represents the unobservable provincial fixed effect, and $\varepsilon_{it}$ is the disturbance term.

Let the weight vector $W={\left(w_{2},\ldots ,w_{J+1}\right)}^{\prime }$, which satisfies *W_j_* ≥ 0 and $w_{2}+\ldots +w_{J+1}=1$, and the outcome variable of synthetic control is expressed as:

(4) $$\overset{J+1}{\underset{j=2}{\sum }}w_{j}E_{jt}=\delta_{t}+\theta_{t}\overset{J+1}{\underset{j=2}{\sum }}w_{j}Z_{j}+\lambda_{t}\overset{J+1}{\underset{j=2}{\sum }}w_{j}\mu_{j}+\overset{J+1}{\underset{j=2}{\sum }}w_{j}\varepsilon_{jt}$$

Suppose there is a weight vector $W^{*}={\left(w_{2}^{*},\ldots ,w_{J+1}^{*}\right)}^{\prime }$, so that:

(5) $$\overset{J+1}{\underset{j=2}{\sum }}w_{j}^{*}E_{j1}=E_{11},\overset{J+1}{\underset{j=2}{\sum }}w_{j}^{*}E_{j2}=E_{12},\ldots ,\overset{J+1}{\underset{j=2}{\sum }}w_{j}^{*}E_{jT_{0}}=E_{1T_{0}}\mathrm{and}\;\overset{J+1}{\underset{j=2}{\sum }}w_{j}^{*}Z_{j}=Z_{1}$$

If $\sum_{t=1}^{T_{0}}\lambda_{t}{}^{\prime }\lambda_{t}$ is nonsingular, the following equation can be obtained:

(6) $$E_{1t}^{N}-\overset{J+1}{\underset{j=2}{\sum }}w_{j}^{*}E_{jt}=\overset{J+1}{\underset{j=2}{\sum }}w_{j}^{*}\overset{T_{0}}{\underset{s=1}{\sum }}\lambda_{t}{\left(\overset{T_{0}}{\underset{n=1}{\sum }}\lambda_{n}{}^{\prime }\lambda_{n}\right)}^{-1}\lambda_{s}{}^{\prime }\left(\varepsilon_{js}-\varepsilon_{1s}\right)-\overset{J+1}{\underset{j=2}{\sum }}w_{j}^{*}\left(\varepsilon_{jt}-\varepsilon_{1t}\right)$$

Abadie et al. [49] proved that the left side of the above formula tends to 0. Therefore, after the implementation of the policy, that is, when *T*_0_ < *t* ≤ *T*, $\sum_{j=2}^{J+1}w_{j}^{*}E_{jt}$ can be used as the unbiased estimation of $E_{1t}^{N}$, and then the estimated value of policy effect $\alpha_{1t}$ can be obtained. The weight W* required for the above estimation is determined by the method proposed by Abadie et al., (2010). *T*_0_ is a time point index, so the number of period variables of [1, *T*_0_] is m. That is, $m=T_{0}-1+1$. Redefining $X_{1}=\left(Z_{1},Y_{1}^{1},\ldots ,Y_{1}^{m}\right)$ as the (*m* × 1) eigenvector for the experimental group in *T*_1_ period, *X_0_* is the (N × m) dimensional correspondence matrix of the control group in T1 period, including *Z_j_* and *Y_jt_* indicators.

Using $∥X_{1}-X_{0}W{∥}_{v}=\sqrt{{\left(X_{1}-X_{0}W\right)}^{\prime }V\left(X_{1}-X_{0}W\right)}$ to measure the distance between $X_{1}$ and $X_{0}W$, and determine $W^{*}$ by minimizing the distance, where V is a (*m* × *m*) positive semidefinite symmetric matrix. The construction of the RMSPE (Root-Mean-Square Percentage Error) statistic can evaluate the deviation degree between the synthetic value ${\hat{Y}}_{1t}$ and the real value $Y_{1t}$ in *t*_1_ period. If RMSPE approaches 0, it indicates that the policy effect ${\overset{\wedge }{gap}}_{1t}$ approaches 0 as a whole, and the synthetic value ${\hat{Y}}_{1t}$ is reliable:

(7) $$RMSPE={\left[\frac{1}{T_{0}-1+1}\overset{T_{0}}{\underset{t=1}{\sum }}{\left(Y_{1t}-{\hat{Y}}_{1t}\right)}^{2}\right]}^{1/2}={\left(\frac{1}{m}\overset{T_{0}}{\underset{t=1}{\sum }}\overset{\wedge }{gap_{1t}^{2}}\right)}^{1/2}$$

### 3.4. Variables Selection

(1) Explained variable

Carbon emission (CE).

In light of the fact that China has not yet published actual carbon emission data, according to the calculation method recommended by the United Nations Intergovernmental Panel on Climate Change (IPCC) in 2006, and on the basis of seven commonly used fossil energy categories, this paper supplemented the collection of ten kinds of energy consumption data, such as washed coal and briquette [50], to calculate the carbon emissions of 30 provinces and cities from 2005 to 2019. The formula is as follows:

(8) $$CE_{ij}=\overset{7}{\underset{i=1}{\sum }}EC_{ij}\times NCV_{ij}\times CC_{i}\times O_{ij}\times \frac{44}{12}$$

where $CE_{ij}$ is the total carbon emission of energy consumption in province *j*, $EC_{ij}$ is the consumption of fuel *i* in province *j*, $NCV_{ij}$, $CC_{i}$ and $O_{ij}$ represent net calorific value, carbon content and oxidation rate, respectively; 44/12 is the molecular ratio of carbon.

Energy efficiency (TFEE).

This paper selects China’s provinces as the decision-making unit to construct the production frontier to calculate the total factor energy efficiency of the production process of each province [51]. As a result, a super efficiency redundancy model with non-radial, non-oriented, VRS (variable return to scale) based and undesired output is constructed.

$$D=\min\frac{1+\frac{1}{m}\sum_{i=1}^{m}s_{i}^{-}/x_{ik}}{1-\frac{1}{q_{1}+q_{2}}\left(\sum_{r=1}^{q_{1}}s_{r}^{+}/y_{rk}+\sum_{t=1}^{q_{2}}s_{t}^{b-}/b_{rk}\right)}$$

$$s.t.\overset{n}{\underset{j=1,j\neq k}{\sum }}x_{ij}\lambda_{j}-s_{i}^{-}\leq x_{ik}$$

$$\overset{n}{\underset{j=1,j\neq k}{\sum }}y_{rj}\lambda_{j}+s_{r}^{+}\geq y_{rk}$$

$$\overset{n}{\underset{j=1,j\neq k}{\sum }}b_{tj}\lambda_{j}+s_{t}^{b-}\leq b_{tk}$$

$$\overset{n}{\underset{j=1,j\neq k}{\sum }}\lambda_{j}=1$$

$$1-\frac{1}{q_{1}+q_{2}}\left(\overset{q_{1}}{\underset{r=1}{\sum }}s_{r}^{+}/y_{rk}+\overset{q_{2}}{\underset{t=1}{\sum }}s_{t}^{b-}/b_{rk}\right)>0$$

$$\lambda ,s^{-},s^{+}\geq 0$$

i=1,2,…,m;r=1,2,…,q;

(9) $$j=1,2,\ldots ,n\left(j\neq k\right)$$

where, *x*, *y* and *b* represent input, expected output and unexpected output variables respectively; $s^{-}$ represents redundant inputs; $s^{+}$ represents insufficient output; λ represents the linear combination coefficient of the decision-making unit. This paper selects the panel data of 30 provincial administrative units in China from 2005 to 2019 (excluding Tibet, Hong Kong, Macao and Taiwan due to lack of data). Among them, the input factors contain capital, labor and energy. The expected output is the total industrial output value, and the unexpected output is the carbon dioxide emission. As the capital data are difficult to obtain directly, they are measured by the net value of fixed assets; labor input is measured by the annual average per capita of all employees; energy input is measured by the total energy consumption of the industrial sector.

(2) Core explanatory variables

Policy dummy variable (Treat × post).

The explanatory variables in this paper are Pilot and Time, which are policy and time dummy variables respectively. Treat × post represents the implementation of the carbon trading pilot policy. In 2013, the carbon emission trading market of pilot provinces and cities gradually began to trade online [52]. Hence, the intersection of policy dummy variables and treatment group is utilized to examine the policy effect. The estimated coefficient measures the effect of the carbon trading pilot policy implemented in 2013 on the emission reduction and energy efficiency of each province. If the coefficient signs of carbon emission and energy efficiency are one negative and one positive, it indicates that the pilot policy of carbon emission trading has effectively facilitated regional emission reduction and energy conservation.

(3) Control variables

Energy structure (ES): fossil energy consumption will bring enormous greenhouse gas emissions. China is rich in coal resources, which also determines its high dependence on coal [53]. This paper adopts the ratio of coal consumption to total energy consumption to represent energy structure. Industrial structure (IND): this study employs the industrial upgrading index obtained by the output value of the tertiary industry divided by the output value of the secondary industry to measure the industrial structure [54]. Energy consumption (EC): this research utilizes the total energy consumption volume to be the proxy for the scale of energy use [55]. Urbanization rate (URB). This paper selects the proportion of urban population in the total population to measure the urbanization rate [56]. Ownership structure (OWN). Under the extensive development mode, China’s state-owned enterprises are more dominated by heavy chemical industry with high energy consumption and large carbon emissions. There may be rent-seeking behavior with the local government, resulting in frequent environmental pollution accidents and becoming an obstacle to energy conservation and emission reduction [57]. Hence, this paper measures the ownership structure by the proportion of state-owned employees in the average number of all employees. Environmental regulation (REGU): this paper utilizes the proportion of pollution control investment to total GDP to measure the intensity of environmental regulation [58]. Research and development (RD): the independent R&D level of each province is expressed by the proportion of R&D expenditure to GDP [59].

### 3.5. Data Source and Feature

**Figure 1:**
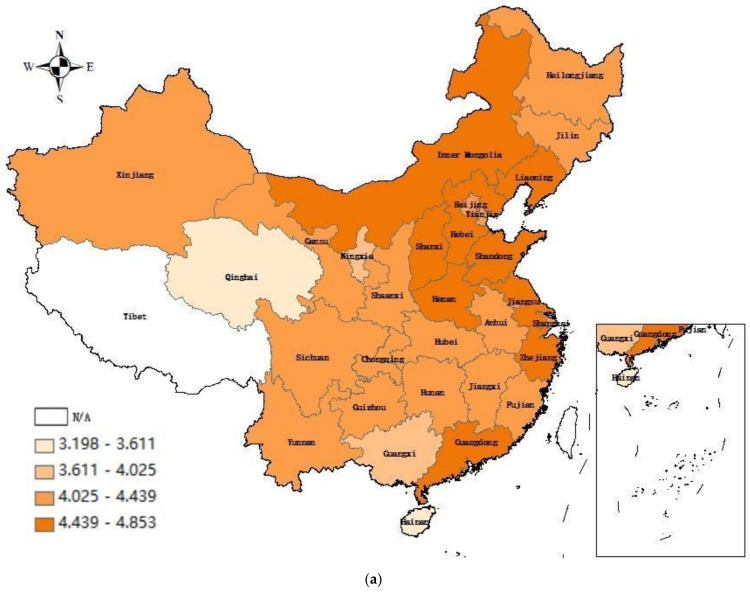

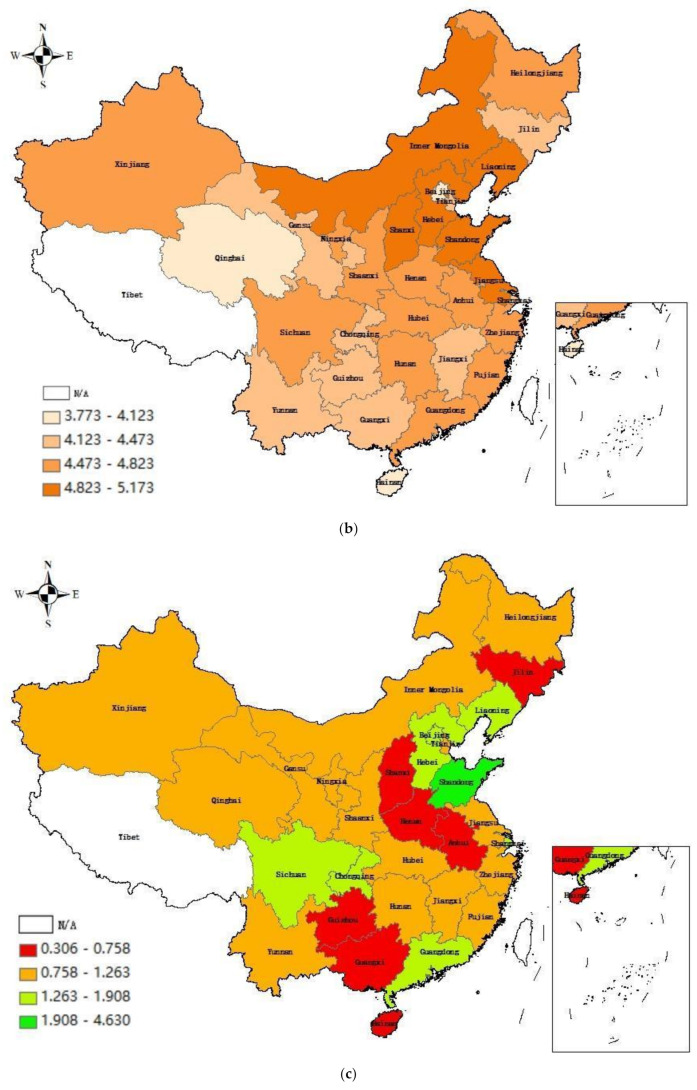

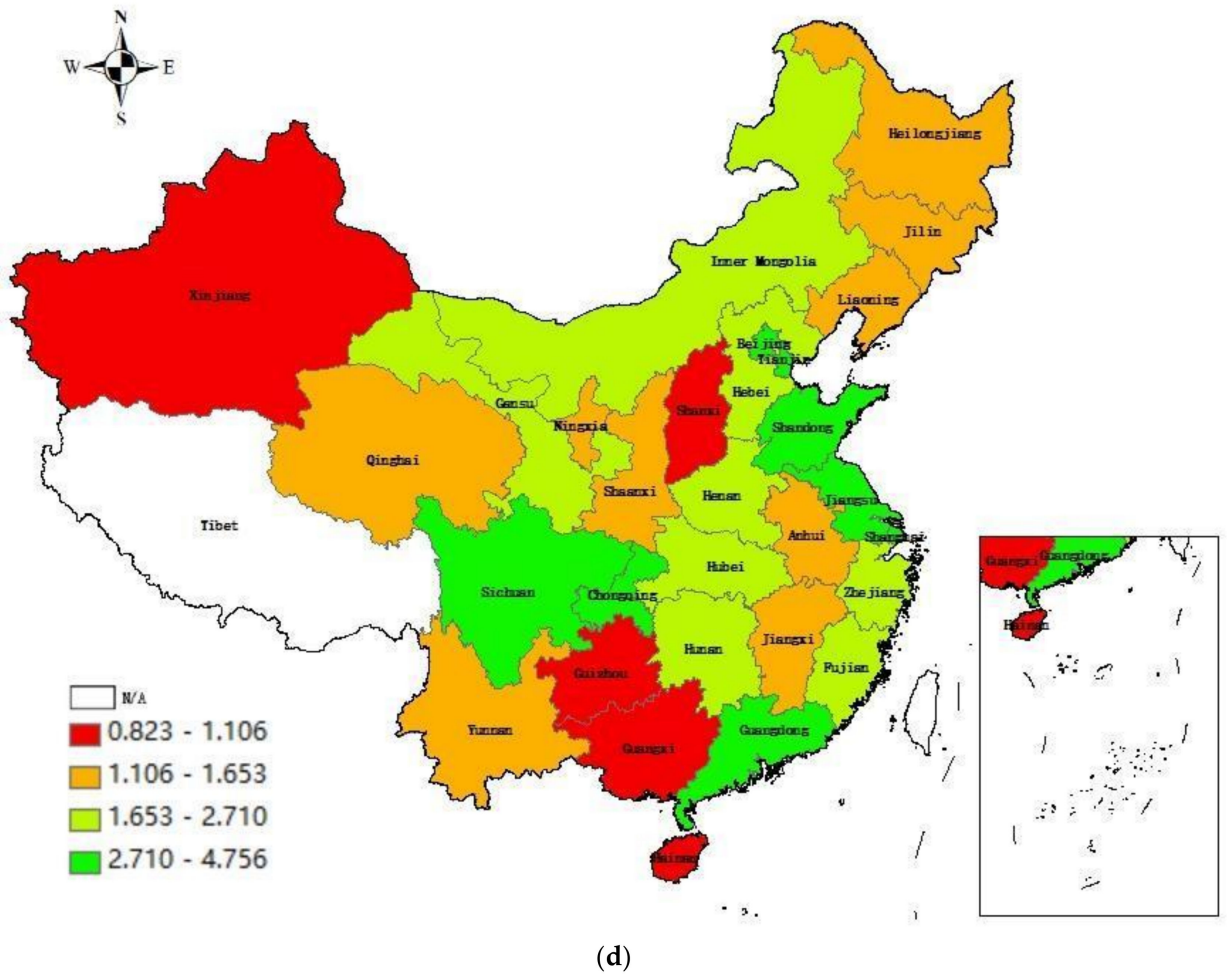
Dynamic change in carbon emissions and TFEE during 2005–2019. (**a**) Provincial carbon emissions in 2005; (**b**) Provincial carbon emissions in 2019; (**c**) Provincial total factor energy productivity in 2005; (**d**) Provincial total factor energy productivity in 2019.

Considering the availability and consistency of data, this paper compiles the panel data of 30 provinces and cities in China from 2005 to 2019 for empirical analysis. Since all the pilot projects except Shenzhen are provinces and municipalities directly under the central government, this study merges Shenzhen into Guangdong Province to unify the research scope [60]. The treatment group in this paper comprises six provinces: Beijing, Tianjin, Shanghai, Chongqing, Hubei and Guangdong. The remaining non-pilot provinces are regarded as the control group. Due to the lack of data, this study excludes Tibet, Hong Kong, Macao and Taiwan. The data stem from China Statistical Yearbook, China Industrial Economic Statistical Yearbook and China Energy Statistical Yearbook. In the meantime, on account of the scientific principle of research samples, this paper uses GDP deflator to convert all monetary quantities into comparable prices via setting 2005 as the base period. This paper employs ArcGIS software to depict the provincial carbon emission and total factor energy productivity map in 2005 and 2019, which are visually manifested in Figure 1a–d. As can be seen from the map, China’s total carbon emissions rose sharply from 2005 to 2019, taking steps on the road to the carbon peak. In the eastern region the main production and living activities of the country are concentrated, so it has always accounted for a large proportion of energy consumption and carbon emissions. In terms of total factor energy productivity, high-productivity areas were mainly concentrated in major urban agglomerations in 2005, such as Beijing–Tianjin–Hebei, Shandong Peninsula, Pearl River Delta, Chengdu-Chongqing. The range of high productivity regions has expanded in 2019, newly joined by the Yangtze River Delta, Central Plains, and Guanzhong Plain. The descriptive statistics of the sample data are listed in Table 1. Compared with the control group, the experimental group has lower carbon emissions and higher total factor energy productivity. Among other variables, energy and industrial structure are also more reasonable, and the level of technological innovation is more advanced. The next empirical section will verify whether this is due to the carbon trading pilot policy having played a role.

## 4. Empirical Research

### 4.1. Baseline Regression

This paper adopts the difference-in-difference (DID) method for empirical analysis of the model (1), and the specific results are exhibited in Table 2. When the province fixed effect, year fixed effect and regional characteristic variables are controlled at the same time, the estimated coefficients of the interaction term treat × post in columns (1) and (2) are significantly negative, demonstrating that the carbon emission trading market can effectively restrict the carbon emission levels of the pilot areas (H1 confirmed). The coefficients of treat × post in columns (3) and (4) are significantly positive, implying that the carbon emission trading market facilitates enhance the energy efficiency of the pilot area (H2 confirmed). Nevertheless, the above analysis only evaluates the average treatment effect of the carbon emission trading market on regional emission reduction and energy efficiency, but does not explain the impact effect of the implementation of the policy on the carbon emission level of pilot provinces and cities over the years. In fact, the carbon emission trading market does not necessarily affect the regional carbon emission levels and energy efficiency in the current period, and its energy saving and emission reduction effect may have a certain lag and sustainability. Hence, this paper introduces the dynamic effect after the year of policy implementation to more rigorously investigate the long-term dynamic impact of the policy on regional emission reduction and energy efficiency.

For columns (5)–(8), the implementation of carbon emission trading market has a long-term significant positive effect on regional energy conservation and emission reduction, and the effect of energy saving and emission reduction has increased year by year with the passage of policy implementation time. The probable reason would be, with the promotion of the construction of carbon emission trading market and the gradual enhancement of relevant policies and supporting measures, the environmental dividend of carbon emission trading market continues to appear, and its impact on regional energy conservation and emission reduction is gradually growing. In addition, after stepwise incorporating control variables into the explanatory variables of the model, the regression results have not changed significantly, which fully demonstrates the robustness of the energy saving and emission reduction effect of the carbon emission trading market. That is, the carbon trading pilot policy has given a substantial boost to regional energy conservation and emission reduction.

### 4.2. Parallel Trend Test

An important assumption for using the DID model to identify the effect of carbon trading pilot policy on energy conservation and emission reduction is that, if there is no impact of carbon trading pilot policy, the development trend of pilot areas (experiment group) and non-pilot areas (control group) should be consistent. The carbon trading pilot policy is proposed by the central government of China and implemented in some regions, its implementation and the designation of pilot areas are rarely affected by local governments. Nevertheless, provinces with larger economic scale, better infrastructure and supporting facilities are more likely to be selected as pilot areas. Thus, there might be differences in energy saving and emission reduction trends between pilot areas and non-pilot areas.

**Figure 2:**
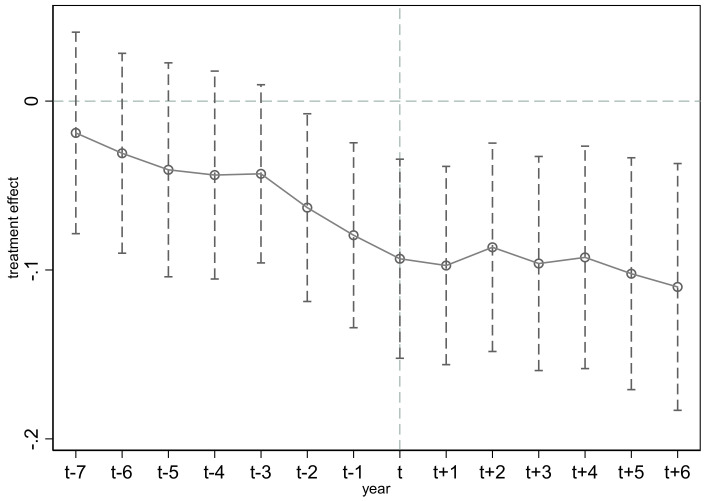
The dynamic effect of the carbon trading pilot policy on carbon emission.

The results in Figure 2 imply that the coefficients of treat × post are not significant before the policy implementation year. That is to say, prior to the launch of the carbon trading pilot policy, the carbon emission difference between the pilot areas and the non-pilot areas is not significant, which is in line with the DID parallel trend hypothesis. The coefficients after the implementation of the policy are significant, which demonstrates that the carbon trading pilot policy does have an influence on carbon emission reduction, and there is no time lag in the impact.

### 4.3. Synthetic Control Method (SCM)

**Figure 3:**
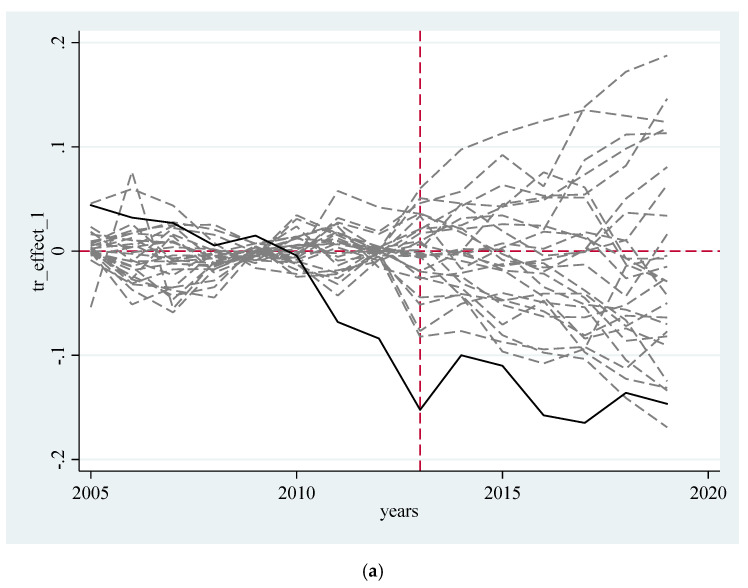

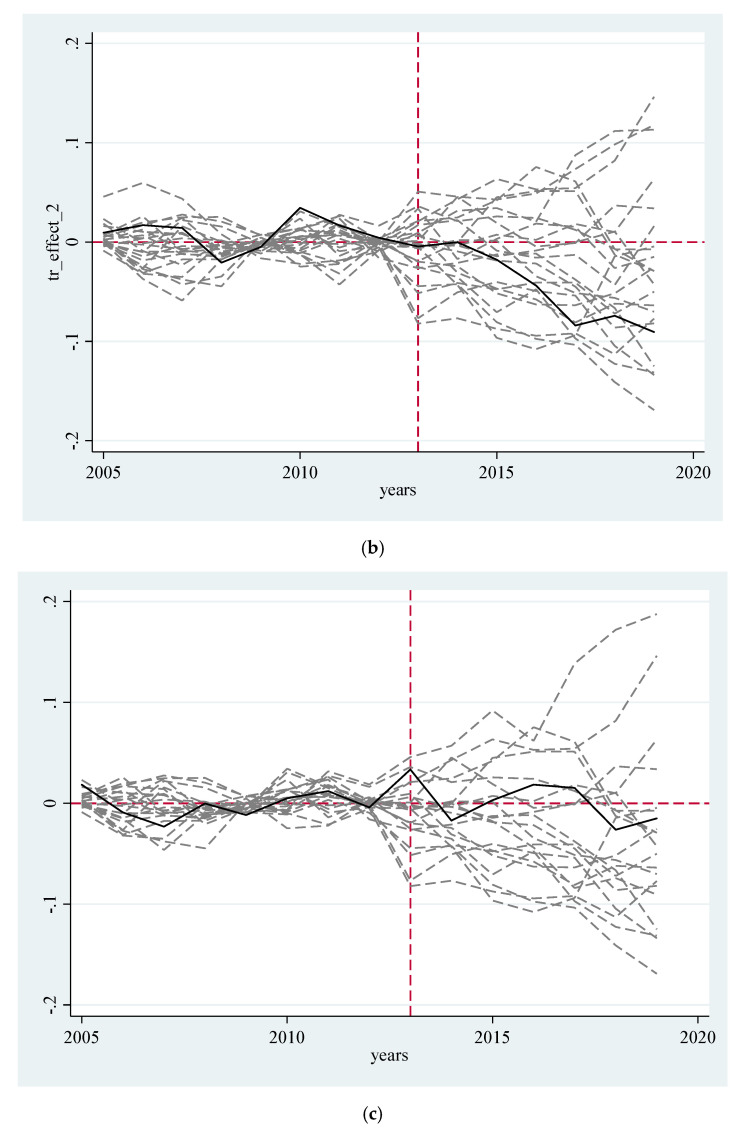

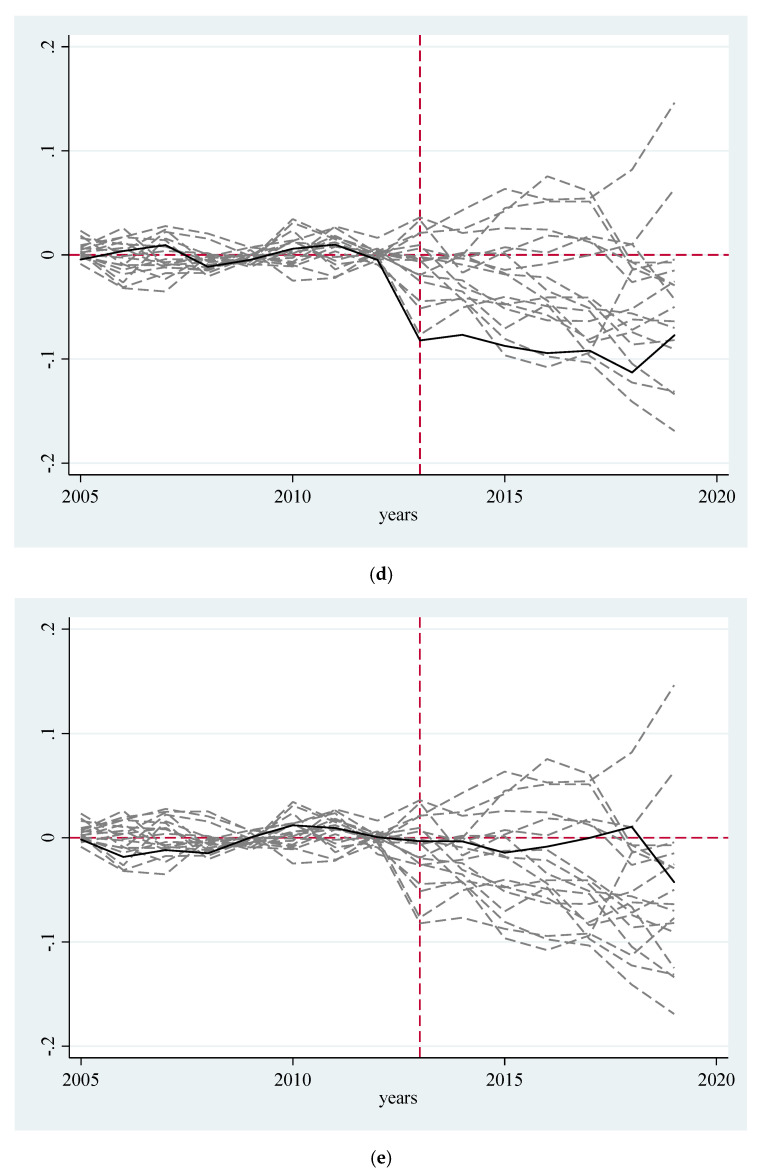

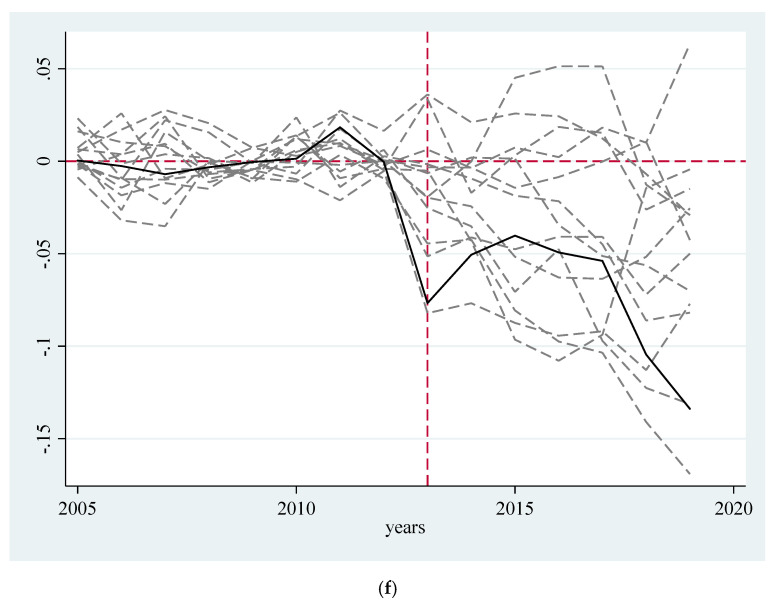
Distribution of carbon emission difference between carbon trading pilot provinces and other provinces. (**a**) Beijing; (**b**) Tianjin; (**c**) Shanghai; (**d**) Hubei; (**e**) Guangdong; (**f**) Chongqing.

This paper adopts root-mean-square prediction errors (RMSPE) to measure the difference in carbon dioxide emissions between carbon trading pilot provinces and their synthetic control groups. To ensure the effect of the ranking test and guarantee the reliability of the simulation of provincial data of the synthetic control group to the data of real provinces after the implementation of the carbon trading pilot policy, this paper deems the implementation year of the carbon trading pilot policy as the dividing point. This paper first calculates the RMSPE value before the implementation of the carbon trading pilot policy, then compare the RMSPE value between the hypothetical pilot province and the real pilot province, and finally eliminate the hypothetical pilot province whose RMSPE value is more than twice that of the real pilot province [61]. If the carbon emissions of the provinces in the synthetic control group cannot better fit the carbon emissions of the policy pilot provinces (containing the hypothetical pilot provinces) before the execution of the carbon emission trading policy, it cannot be guaranteed that the differences after the implementation of the pilot policy are caused by the implementation of the policy. As is arrayed in Figure 3, the solid line represents the corresponding pilot provinces, and the dotted line refers to other provinces whose RMSPE value was less than twice that of the pilot provinces before 2013. The left side of the vertical dotted line is the difference between the carbon emissions of the disposal object and its synthetic object before 2013, and the right side is the difference after 2013. Beijing, Tianjin, Shanghai, Hubei, Guangdong and Chongqing have a large gap with the carbon dioxide emissions of synthetic cities with probabilities of 3.7% (1/27), 4% (1/25), 4.7% (1/21), 5.5% (1/18), 5.2% (1/19) and 7.1% (1/14) respectively. Therefore, it can be considered that the assumption of the impact of the carbon trading pilot policy on the carbon emission reduction of these pilot provinces is not caused by other accidental factors, at least at the significance level of 10%.

The DID estimation method requires that the experimental group and the control group can be compared before the execution of the policy, but the pilot areas often have peculiarity, which may lead to endogenous problems of the policy. The synthetic control method constructs a “counterfactual” reference group through the weighted average of other provinces and cities, which can avoid the endogenous problem [62]. In order to ensure the reliability of this research conclusion, next, this paper adopts the synthetic control method to test the robustness. Figure 3 presents the simulation results with Beijing, Tianjin, Shanghai, Hubei, Guangdong and Chongqing as experimental groups. According to the results, it can be found that before the implementation of the carbon emission trading system, the energy consumption and carbon dioxide emission paths of the real pilot provinces and cities are similar to those of the synthetic pilot provinces and cities. After the implementation of the carbon emission trading system, the energy consumption and carbon dioxide emission of the real pilot provinces and cities are significantly lower than those of the synthetic pilot provinces and cities, and the gap between most pilot provinces (cities and autonomous regions) is gradually widening. Therefore, the fitting results based on the synthetic control method verify the robustness of the research conclusions in this paper.

### 4.4. Propensity Scoring Matching (PSM)

In order to avoid possible sample selectivity errors, the propensity score matching method (PSM) is used to alleviate this problem, and then the DID method is performed to estimate the impact of the carbon trading pilot policy on energy conservation and emission reduction. The DID method has a certain subjectivity in the selection of the control group, especially when there are systematic differences between the treatment group and the control group. It will have an impact on the robustness and credibility of the evaluation results. Consequently, this paper further adopts the PSM–DID method to eliminate the systematic differences in characteristics between the two groups by matching the individuals of the treatment group and the control group in the light of their similarity, and then performs regression analysis. Firstly, the observable variables are selected to match the experimental group and the control group. The Logit model is used to calculate the propensity score of the above matching indicators, and then the matching method is used to match the provincial samples of the experimental group and the control group, excluding the samples with unsuccessful matching. The stationarity test of each variable before and after propensity score matching is displayed in Table 3.

It can be seen that the bias of variables has decreased compared with that before matching, illustrating that there is no significant difference between the experimental group and the control group, and the distribution has become more balanced. Therefore, this method is more appropriate. The *p*-value of all variables after matching is greater than 0.1, implying that there is no significant difference between the experiment group and the control group after matching. Moreover, after matching, there is no absolute value of bias that is greater than 10%, which demonstrates that the characteristics of the experimental group and the control group after matching are very close, thus meeting the stationarity hypothesis of PSM.

Using the Logit model and taking the explanatory variables as the corresponding matching variables, the control variable group is matched with propensity score for the experimental group and the control group, and then the methods of kernel matching, radius matching and k-nearest neighbor matching are used for sample matching, respectively. On this basis, the DID method is employed to identify the net impact of the carbon trading pilot policy on regional carbon emission level and energy efficiency. In principle, the final regression results do not depend on the selection of specific matching methods. By carrying out regression analysis on the matched data again, and the PSM–DID estimation results are shown in Table 4.

Table 4 displays the estimation results after using different matching methods. When taking the carbon trading pilot as the dependent variable, the regression coefficients of policy variable in the kernel matching results displayed in columns (1) and (4) are −0.098 and 0.495, and passed the 5% significance level test. This signifies that when the emission trading system is established, carbon emissions will be suppressed and energy efficiency will be improved. On the whole, the carbon trading pilot has promoted green development, and the two have a causal relationship. The results of k-neighbor matching and radius matching are also consistent with this. It verifies the applicability and feasibility of the PSM model for the environmental effect variables of the carbon trading pilot policy. Namely, the carbon trading system is conducive to emission reduction and energy saving (elevation of energy efficiency) across the pilot areas (H3 confirmed), indicating the robustness of the baseline regression results.

### 4.5. Placebo Test

**Figure 4:**
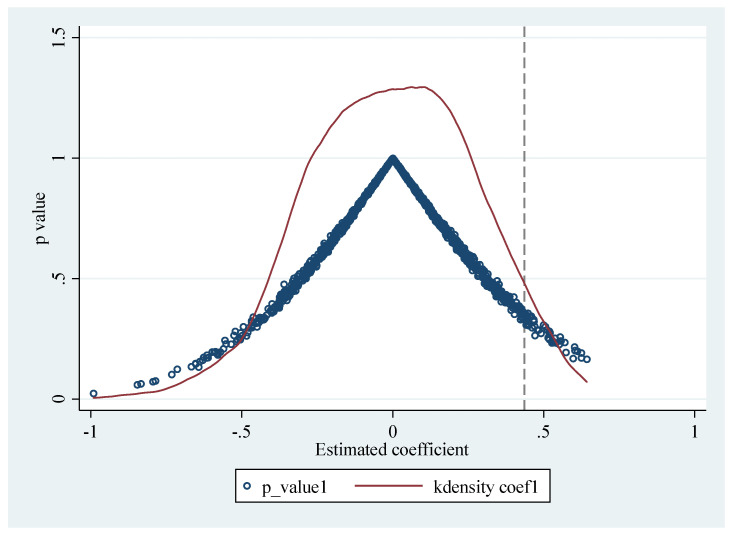
Kernel density estimate.

To further eliminate the differences in energy conservation and emission reduction between pilot and non-pilot provinces caused by other multiple composite factors, this paper constructs a false treatment group and control group for placebo test [63]. From the total sample of 30 provinces, six provinces are randomly selected as the treatment group and other provinces as the control group. Through random sampling, the groups of false carbon trading policy pilot provinces and non-pilot provinces are constructed. The dummy variable of false carbon trading policy pilot provinces is assigned as 1 and the virtual variable of false non-pilot provinces is assigned as 0. Then, this paper conducts the benchmark model regression with provincial characteristic variables and year control variables, repeat the random sampling for 400 times, and draw the distribution map of regression coefficient kernel density in Figure 4.

As can be observed from Figure 4, in the baseline model estimation results of the samples of virtual pilot provinces and virtual non-pilot provinces constructed by random sampling, in comparison to the baseline regression result of 0.436, the coefficients are distributed around 0. It demonstrates that, in the case of using the constructed false data of pilot provinces and non-pilot provinces, energy conservation and emission reduction at the provincial level have not been significantly affected. In other words, the estimation results of the benchmark model in this paper have passed the placebo test, which further proves that the enhancement of the effect of energy saving and emission control in the pilot provinces is caused by the carbon trading pilot policy.

## 5. Conclusions

Based on the panel data of 30 provinces from 2005 to 2019, this paper probes the impact of carbon trading pilot policy on total carbon emissions and energy efficiency based on the difference in difference (DID) and synthetic control method (SCM). The main research results are listed below. (1) The carbon trading mechanism is conducive to reducing total carbon emissions and improving total factor energy productivity, but the emission reduction effects of each pilot area are different. (2) The emission reduction effect of the carbon trading mechanism in Tianjin and Shanghai is the best, and the emission reduction effect on total carbon emissions remains stable; (3) The emission reduction effect of the carbon trading mechanism in Guangdong exhibits a strong to weak trend; (4) The carbon trading mechanism mainly produces emission reduction effect by optimizing industrial structure and uplifting energy efficiency. First, enterprises eliminate backward production capacity under cost pressure and propel the flow of production factors to enterprises in technology intensive industries, so as to adjust the economic structure and alleviate carbon emissions [64,65]. Secondly, under the cost pressure and the driving force of emission reduction technology innovation, enterprises tend to improve emission reduction technology to advance energy efficiency and finally achieve the goal of energy conservation and emission reduction [66,67].

## 6. Policy Implications

The findings underline the role of carbon trading mechanism in reducing the total amount of emission reduction and improving energy efficiency, which can not only become a strong support for the transformation of China’s economic incentive environmental policy tools, but also accumulate valuable experience for carbon trading across the country, and provide a practical basis for the dynamic adjustment and correction of carbon trading system design [68]. The policy implications of this paper are reflected in the next several aspects.

(1) Strengthen government guidance and make full use of the promotion effect of carbon trading market on technological innovation. The government should provide a platform for enterprises to stimulate the efficiency of R&D and emission reduction, impel enterprises to elevate the efficiency of technology transfer, and construct a rewarding environment for R&D and technological innovation [69]. Support innovative leading enterprises in the carbon trading market, strengthen publicity, and create a sound atmosphere for technological innovation in energy conservation and emission reduction. For innovative enterprises and institutions with insufficient funds, it is important to augment the support of green finance, comprising tax, subsidy, transfer payment, credit and other support. Besides this, considering the dynamic evolution of the impact of carbon trading on energy conservation and emission reduction, the government should establish a monitoring and feedback mechanism to continuously track and follow up the emission control efforts and energy efficiency in various regions. In the process of building a national carbon emissions trading market, attention should be paid to regional differences, and careful progress should be made on the basis of systematically summarizing the pilot experience of carbon emissions trading. The government should successfully complete the role transition, fully coordinate the relationship between environmental policy tools and give play to their regional synergy.
(2) Establish comprehensive public policy and industrial policy system that encourage the transformation of energy structure and industrial structure. On the one hand, in terms of increasing the R&D and innovation of basic originality and common technologies in the new energy industry, the government should play a greater role in the investment of basic R&D funds and the construction of common platforms for the formation of R&D achievements [70,71]. Since TFEE is the key to realize energy conservation and efficiency increase, it is necessary to promote the rational cross regional allocation of renewable energy and technology spillover effect. Encourage and lead the energy consumption structure change from fossil energy to clean energy such as renewable energy, on the premise that the technology of renewable energy is stable and the cost is reduced. On the other hand, the government needs to actively develop strategic emerging industries, spur the integration of the achievements of the new generation of information technology (Internet of Things, big data and artificial intelligence) with traditional industries, and realize the low carbonization of the industrial system.
(3) Accelerate the development of service industry and expand the proportion of clean energy consumption. The 14th Five-year Plan is a critical and window period for reaching the carbon peak. At present, China vigorously advocates supply side structural reform and eliminates backward production capacity, which highlighting the importance of industrial structure adjustment to emission abatement and energy conservation [72,73]. While achieving the emission reduction target, it is also necessary to stimulate the initiative of market players, enhance the optimal allocation of low-carbon elements, and optimize the energy structure. At the same time, with the control of carbon emissions as a constraint means, enterprises are encouraged to carry out technological transformation and upgrading to resolve high-carbon backward production capacity. For the current stage, China’s carbon market practice has proved that carbon trading can derive green consumption demand, and then force the optimization of industrial structures, strengthen the proportion of clean industry, and build a clean, low-carbon, safe and efficient energy system. In the meantime, enterprises should gradually transition from relying on energy consumption to relying on human capital and R&D investment to raise output, thereby realize the upgrading of factor structure and advance the efficiency of factor resource allocation.
(4) Ameliorate the market incentive mechanism for the transformation of scientific and technological achievements, and accelerate the green transition and upgrading of enterprises. Relying on the carbon emission trading policy and the national online carbon trading market, proactively guide universities and scientific research institutions to explore green total factor productivity, and continue to implement the long-term mechanism of in-depth integration and development of IUR (industry, university and research institute). In addition, the government should encourage, support and guide enterprises to engage in green innovation, achievement transformation and green transformation and development, explore the best scheme conducive to the growth of green total factor productivity by supporting R&D, subsidies, tax rebates, exemptions, providing loan guarantees and patent authorization, and give full play to the synergistic governance role of market leadership and government guidance [74]. Eventually, the goal should be to form a market environment beneficial to the development of enterprise technological innovation and industrial structure transformation in the direction of greening. It is needed to improve the design of carbon trading mechanisms, strengthen supervision, help improve the carbon trading market, and boost the innovation ability of low-carbon technology. Strengthening the management of policy signals and elevating the openness, sustainability and consistency of the central and local carbon emission trading policies will stabilize the expectations of enterprises and the public and guide the implementation of low-carbon technology innovation activities.
(5) Enterprises should strengthen the R&D of energy saving and emission reduction technologies, accumulate relevant R&D talents. In detail, they should cooperate with professional research institutions in R&D and patent purchase, actively participate in the carbon trading market and obtain income through the sale of shares, thus form a long-term benign interaction with energy saving and emission reduction technology innovation [75]. They should also enhance the innovative awareness of energy conservation and emission reduction, accurately measure the costs and benefits of innovation, and make reasonable long-term decisions. Simultaneously, it is necessary to strengthen the management level of enterprises and the use efficiency of funds, take advantage of the support of national policies and funds, and facilitate their own technological innovation ability. China’s low-carbon pilot areas show different emission reduction characteristics due to varied conditions. Therefore, the low-carbon transformation should encourage local enterprises to form a distinctive geographical layout, industrial system, production and life mode according to the regional economic development stage and resource situation.

## Figures and Tables

**Table 1:** Descriptive statistics of the variables.

Variable	Variable Name	CE	TFEE	ES	IND	EC	URB	OWN	REGU	RD	Numbers
All samples	Mean	4.462	1.434	42.887	1.073	4.033	54.532	27.586	0.149	1.475	450
	Std. Dev.	0.332	0.8251	15.529	0.606	0.305	14.017	13.192	0.131	1.081	
	Minimum	3.197	0.306	1.213	0.381	2.913	26.87	5.821	0.002	0.18	
	Maximum	5.173	4.756	76.005	5.169	4.616	89.6	134.671	0.991	6.31	
Experimentalgroup	Mean	4.367	1.954	31.135	1.552	4.026	71.897	18.617	0.096	2.792	90
	Std. Dev.	0.235	0.879	14.936	1.024	0.233	14.898	8.633	0.082	1.526	
	Minimum	4.029	0.852	1.21	0.727	3.614	43.2	5.938	0.002	1.04	
	Maximum	4.837	4.631	62.61	5.169	4.533	89.6	42.138	0.503	6.31	
Control group	Mean	4.486	1.304	45.825	0.954	4.035	50.191	29.829	0.162	1.146	360
	Std. Dev.	0.348	0.758	14.249	0.357	0.321	9.809	13.191	0.138	0.582	
	Minimum	3.197	0.306	8.37	0.381	2.913	26.87	5.821	0.011	0.18	
	Maximum	5.173	4.756	76.01	2.847	4.616	72.47	134.671	0.991	2.79	

**Table 2:** Baseline regression results.

Variable	Average Treatment Effect				Long-Run Dynamic Effect			
	(1) CE	(2) CE	(3) TFEE	(4) TFEE	(5) CE	(6) CE	(7) TFEE	(8) TFEE
treat × post	−0.092 * (1.21)	−0.008 * (0.38)	0.567 *** (3.24)	0.464 *** (3.03)				
pilot × time2013					−0.126 *** (−3.49)	−0.081 *** (−3.80)	0.544 *** (2.11)	0.436 * (1.47)
pilot × time2014					−0.133 *** (−3.74)	−0.086 *** (−4.20)	0.547 *** (2.11)	0.453 * (1.50)
pilot × time2015					−0.131 *** (−3.65)	−0.087 *** (−3.85)	0.529 *** (2.02)	0.433 * (1.37)
pilot × time2016					−0.142 *** (−3.83)	−0.102 (−4.42)	0.554 *** (2.10)	0.489 ** (1.57)
pilot × time2017					−0.148 *** (−3.85)	−0.103 *** (−4.12)	1.181 *** (3.62)	1.124 *** (3.10)
pilot × time2018					−0.162 *** (−4.07)	−0.101 *** (−4.07)	1.399 *** (4.47)	1.312 *** (3.47)
pilot × time2019					−0.172 *** (−4.22)	−0.112 *** (−3.93)	1.905 *** (5.11)	1.829 *** (4.20)
ES		0.002 *** (5.263)		−0.015 *** (−4.918)		0.001 ** (1.85)		−0.007 *** (−3.52)
IND		0.037 *** (3.478)		0.038 (0.481)		−0.027 *** (−2.02)		0.209 *** (2.15)
EC		1.036 *** (58.75)		1.079 *** (8.382)		1.002 *** (13.38)		−0.626 *** (−2.07)
URB		0.005 *** (8.818)		−0.011 *** (−2.341)		−0.002 *** (−2.28)		0.022 *** (2.29)
OWN		0.001 ** (1.693)		−0.003 (−0.815)		−0.001 *** (−2.51)		0.008 *** (3.55)
REGU		0.245 *** (6.847)		0.153 (0.585)		0.032 (1.41)		−0.075 (−0.53)
RD		−0.043 *** (−5.715)		0.219 *** (4.032)		−0.006 (−0.65)		0.157 (1.15)
Constant	4.469 *** (265.60)	−0.121 (−1.01)	1.330 *** (36.53)	−2.134 *** (−3.50)	4.429 *** (184.56)	−0.114 (−0.98)	1.084 *** (22.91)	−1.999 *** (−3.42)
Time effect	YES	YES	YES	YES	YES	YES	YES	YES
Province effect	YES	YES	YES	YES	YES	YES	YES	YES
R−squared	0.06	0.93	0.20	0.42	0.97	0.99	0.91	0.92

Note: * *p* < 0.1. ** *p* < 0.05. *** *p* < 0.01. The values in the brackets are t-statistics.

**Table 3:** Stationary test of variables before and after matching.

Variables	Unmatched/Matched	Mean Value		Bias(%)	Test	
		Experiment	Control		t-Value	*p*-Value
ES	U	31.136	45.825	−100.6	−8.66	0.000
	M	39.638	38.826	5.6	0.29	0.770
IND	U	1.5521	0.9541	78.0	9.10	0.000
	M	0.9918	1.0126	−2.7	−0.39	0.699
EC	U	4.0269	4.0354	−3.0	−0.24	0.813
	M	4.1463	4.1505	−1.5	−0.08	0.940
URB	U	71.898	50.191	172.1	16.73	0.000
	M	59.026	59.318	−2.3	−0.16	0.875
OWN	U	18.618	29.829	−100.6	−7.66	0.000
	M	19.624	20.386	−6.8	−0.31	0.756
REGU	U	0.0961	0.1626	−58.7	−4.39	0.000
	M	0.0972	0.0998	−2.3	−0.17	0.866
RD	U	2.7927	1.1461	142.5	16.30	0.000
	M	1.7165	1.728	−1.0	−0.09	0.925

**Table 4:** PSM–DID test results.

Variable	CE			TFEE		
	(1) Kernel Matching	(2) Radius Matching	(3) K-Neighbor Matching	(4) Kernel Matching	(5) Radius Matching	(6) K-Neighbor Matching
treat × post	−0.098 *** (−1.62)	−0.077 *** (−1.13)	−0.090 *** (−1.36)	0.495 *** (3.06)	0.493 *** (2.51)	0.519 *** (2.99)
Constant	−1.861 (−0.41)	−1.861 (−0.41)	−1.861 (−0.41)	−1.861 (−0.41)	−1.861 (−0.41)	−1.861 (−0.41)
Control variables	YES	YES	YES	YES	YES	YES
Time fixed	YES	YES	YES	YES	YES	YES
Province fixed	YES	YES	YES	YES	YES	YES
R-squared	0.588	0.588	0.588	0.588	0.588	0.588

Note: *** *p* < 0.01. The values in the brackets are t-statistics.

## Data Availability

The data used to support the findings of this study are available from the corresponding author upon request.
